# Creatinium hydrogen oxalate

**DOI:** 10.1107/S1600536812000852

**Published:** 2012-01-14

**Authors:** A. Jahubar Ali, S. Athimoolam, S. Asath Bahadur

**Affiliations:** a Department of Science and Humanities, National College of Engineering, Maruthakulam, Tirunelveli 627 151, India; b Department of Physics, University College of Engineering Nagercoil, Anna University of Technology Tirunelveli, Nagercoil 629 004, India; c Department of Physics, Kalasalingam University, Anand Nagar, Krishnan Koil 626 190, India

## Abstract

The crystal structure of the title compound, C_4_H_10_N_3_O_2_^+^·C_2_HO_4_^−^, is stabilized by N—H⋯O and O—H⋯O hydrogen bonds. The anions are connected by an O—H⋯O hydrogen bond, leading to *C*(5) chain extending along *c* axis. The cations are dimerized around the corners of the unit cell, leading to an *R*_2_^2^(14) ring motif. This leads to a cationic mol­ecular aggregation at *x* = 0 or 1 and an anionic mol­ecular aggregation at *x* = 1/2.

## Related literature

For related structures see: Ali *et al.* (2011*a* ▶,*b* ▶); Bahadur, Kannan *et al.* (2007 ▶); Bahadur, Sivapragasam *et al.* (2007 ▶); Bahadur, Rajalakshmi *et al.* (2007 ▶). For hydrogen-bonding motifs, see Bernstein *et al.* (1995 ▶). For the biological importance of creatine, see: Cannan & Shore (1928 ▶); Greenhaff *et al.* (1993 ▶).
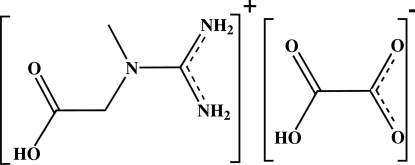


## Experimental

### 

#### Crystal data

C_4_H_10_N_3_O_2_^+^·C_2_HO_4_^−^
*M* *_r_* = 221.18
Monoclinic, 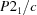

*a* = 7.1545 (4) Å
*b* = 12.3681 (7) Å
*c* = 10.5151 (6) Å
β = 94.18 (1)°
*V* = 927.98 (9) Å^3^
*Z* = 4
Mo *K*α radiation
μ = 0.14 mm^−1^
*T* = 293 K
0.24 × 0.22 × 0.18 mm

#### Data collection

Bruker SMART APEX CCD area-detector diffractometer
8631 measured reflections
1641 independent reflections
1587 reflections with *I* > 2σ(*I*)
*R*_int_ = 0.018

#### Refinement

*R*[*F* ^2^ > 2σ(*F* ^2^)] = 0.037
*wR*(*F* ^2^) = 0.102
*S* = 1.08
1641 reflections
162 parameters
1 restraint
H atoms treated by a mixture of independent and constrained refinement
Δρ_max_ = 0.25 e Å^−3^
Δρ_min_ = −0.23 e Å^−3^

### 

Data collection: *SMART* (Bruker, 2001 ▶); cell refinement: *SAINT* (Bruker, 2001 ▶); data reduction: *SAINT*; program(s) used to solve structure: *SHELXTL/PC* (Sheldrick, 2008 ▶); program(s) used to refine structure: *SHELXTL/PC*; molecular graphics: *PLATON* (Spek, 2009 ▶); software used to prepare material for publication: *SHELXTL/PC*.

## Supplementary Material

Crystal structure: contains datablock(s) global, I. DOI: 10.1107/S1600536812000852/hg5159sup1.cif

Structure factors: contains datablock(s) I. DOI: 10.1107/S1600536812000852/hg5159Isup2.hkl

Supplementary material file. DOI: 10.1107/S1600536812000852/hg5159Isup3.cml

Additional supplementary materials:  crystallographic information; 3D view; checkCIF report

## Figures and Tables

**Table 1:** Hydrogen-bond geometry (Å, °)

*D*—H⋯*A*	*D*—H	H⋯*A*	*D*⋯*A*	*D*—H⋯*A*
N2—H1*N*⋯O1^i^	0.82 (3)	2.37 (3)	3.094 (2)	148 (2)
N2—H2*N*⋯O11^ii^	0.85 (2)	2.08 (2)	2.910 (2)	168 (2)
N3—H3*N*⋯O1^iii^	0.85 (2)	2.18 (2)	2.985 (2)	157 (2)
N3—H4*N*⋯O14	0.86 (2)	2.04 (2)	2.903 (2)	174 (2)
O2—H2⋯O12^iv^	0.94 (3)	1.60 (3)	2.538 (2)	173 (2)
O13—H13*O*⋯O11^v^	0.90 (3)	1.72 (3)	2.605 (1)	168 (2)

Symmetry codes: (i) 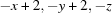; (ii) 
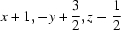
; (iii) 
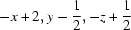
; (iv) 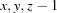; (v) 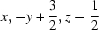.

**Fig. 1.**
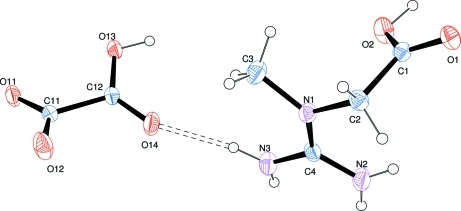
The molecular structure of the title compound (I) with the numbering scheme for the atoms and 50% probability displacement ellipsoids. H bonds are drawn as dashed lines.

**Fig. 2.**
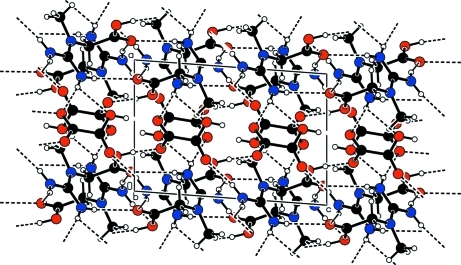
Packing diagram of the molecules viewed down the b-axis. H atoms not involved in the H-bonds (dashed lines) are omitted for clarity.

## supplementary crystallographic information

### Comment

Creatine is a nitrogenous organic acid that occurs naturally in vertebrates and helps to supply energy to all cells in the body, primarily muscle (Cannan, 1928; Greenhaff *et al.*,1993)). We are interested in the specificity of recognition between organic acids and cretine and creatinine molecules and have reported a number of creatinine related structures (Ali *et al.*, 2011*a*,b; Bahadur, Kannan *et al.*, 2007; Bahadur, Sivapragasam *et al.*, 2007; Bahadur, Rajalakshmi *et al.*, 2007).

The asymmetric part of the title compound, (I), contains one creatinium cation and one hydrogen oxalate anion (Fig. 1). The protonation of the N site of the cation is evident from C—N bond distances. The deprotonation on the one of the –COOH groups of the oxalic acid is confirmed from that –COO^-^ bond geometry. The planes of –COOH and –COO^-^ groups are twisted out from each other with an angle of 25.1 (2)°. This twisting of planes may be caused due to the hydrogen bonding association and molecular aggregation. The crystal structure and the molecular aggregations are stabilized through intricate three dimensional hydrogen bonding network (Fig. 2; Table 1). All the N and O atoms of the cation and anion participate in the hydrogen bonding interactions.

Hydrogen oxalate anions are connected themselves through a O—H···O hydrogen bond leading to a linear chain C(5) motif extending along *c* axis of the unit cell (Bernstein *et al.*, 1995). Creatinium cations are dimerized around inversion centres of the unit cell, especially at the corners of the unit cell and making a ring *R*_2_^2^(4) motif through N2—H1N···O1 (2 - *x*, 2 - *y*, -*z*) hydrogen bond. Also, these cationic dimers are connected themselves through another N—H···O hydrogen bond leading to a zigzag chain C(7) motif extending along *b* axis of the unit cell [N3—H3N···O1(-*x* + 2, *y* - 1/2, -*z* + 1/2)]. These interconnected cationic dimers are connected with oxalate anion leading to a zigzag chain C_2_^2^(11) motif extending along *ac*-plane of the unit cell through N2—H2N···O11(1 + *x*, 3/2 - *y*, -1/2 + *z*) and O2—H2···O12(*x*, *y*, -1 + *z*). Another pair of N—H···O and O—H···O hydrogen bonds between cation and anion leading to a linear chain C_2_^2^(12) motifs extending along *c* axis of the unit cell [N3—H3N···O14 and O2—H2···O12(*x*, *y*, -1 + *z*)]. Dimerization of cations and anionic chain motifs lead to cationic molecular aggregation at *x*=0 or 1 and molecular aggregation of anions at *x*=1/2. These cationic and anionic aggregations are connected further through other N—H···O hydrogen bonds leading to a three dimensional hydrogen bonding network.

### Experimental

The title compound was crystallized from an aqueous mixture containing creatine (0.13g) and oxalic acid (0.09g) in the stoichiometric ratio of 1:1 (20 ml of water) at room temperature by slow evaporation technique.

### Refinement

All the H atoms except the atoms involved in hydrogen bonds were positioned geometrically and refined using a riding model, with C—H = 0.96 (–CH_3_) and 0.97 Å (–CH_2_) and *U*_iso_(H) = 1.2–1.5 *U*_eq_ (parent atom). H atoms involved in hydrogen bonds were located from differential Fourier maps and refined isotropically.

### Crystal data

**Table:**

C_4_H_10_N_3_O_2_^+^·C_2_HO_4_^−^	*F*(000) = 464
*M**_r_* = 221.18	*D*_x_ = 1.583 Mg m^−^^3^
Monoclinic, *P*2_1_/*c*	Mo *K*α radiation, λ = 0.71073 Å
Hall symbol: -P 2ybc	Cell parameters from 3216 reflections
*a* = 7.1545 (4) Å	θ = 2.1–24.7°
*b* = 12.3681 (7) Å	µ = 0.14 mm^−^^1^
*c* = 10.5151 (6) Å	*T* = 293 K
β = 94.18 (1)°	Block, colourless
*V* = 927.98 (9) Å^3^	0.24 × 0.22 × 0.18 mm
*Z* = 4	

### Data collection

**Table:**

Bruker SMART APEX CCD area-detector diffractometer	1587 reflections with *I* > 2σ(*I*)
Radiation source: fine-focus sealed tube	*R*_int_ = 0.018
graphite	θ_max_ = 25.0°, θ_min_ = 2.6°
ω scans	*h* = −8→8
8631 measured reflections	*k* = −14→14
1641 independent reflections	*l* = −12→12

### Refinement

**Table:**

Refinement on *F*^2^	Secondary atom site location: difference Fourier map
Least-squares matrix: full	Hydrogen site location: inferred from neighbouring sites
*R*[*F*^2^ > 2σ(*F*^2^)] = 0.037	H atoms treated by a mixture of independent and constrained refinement
*wR*(*F*^2^) = 0.102	*w* = 1/[σ^2^(*F*_o_^2^) + (0.0629*P*)^2^ + 0.2614*P*] where *P* = (*F*_o_^2^ + 2*F*_c_^2^)/3
*S* = 1.08	(Δ/σ)_max_ < 0.001
1641 reflections	Δρ_max_ = 0.25 e Å^−^^3^
162 parameters	Δρ_min_ = −0.23 e Å^−^^3^
1 restraint	Extinction correction: *SHELXTL/PC* (Sheldrick, 2008), Fc^*^=kFc[1+0.001xFc^2^λ^3^/sin(2θ)]^-1/4^
Primary atom site location: structure-invariant direct methods	Extinction coefficient: 0.040 (6)

### Special details

**Table:**

Geometry. All esds (except the esd in the dihedral angle between two l.s. planes) are estimated using the full covariance matrix. The cell esds are taken into account individually in the estimation of esds in distances, angles and torsion angles; correlations between esds in cell parameters are only used when they are defined by crystal symmetry. An approximate (isotropic) treatment of cell esds is used for estimating esds involving l.s. planes.
Refinement. Refinement of *F*^2^ against ALL reflections. The weighted *R*-factor *wR* and goodness of fit *S* are based on *F*^2^, conventional *R*-factors *R* are based on *F*, with *F* set to zero for negative *F*^2^. The threshold expression of *F*^2^ > σ(*F*^2^) is used only for calculating *R*-factors(gt) *etc*. and is not relevant to the choice of reflections for refinement. *R*-factors based on *F*^2^ are statistically about twice as large as those based on *F*, and *R*- factors based on ALL data will be even larger.

### Fractional atomic coordinates and isotropic or equivalent isotropic displacement parameters (Å^2^)

**Table:**

	*x*	*y*	*z*	*U*_iso_*/*U*_eq_	
C1	0.83544 (18)	1.03293 (11)	0.10352 (12)	0.0299 (3)	
C2	0.92061 (19)	1.05574 (11)	0.23633 (13)	0.0326 (3)	
H2A	1.0542	1.0671	0.2321	0.039*	
H2B	0.8676	1.1225	0.2661	0.039*	
C3	0.7146 (2)	0.97346 (13)	0.38693 (16)	0.0440 (4)	
H3A	0.6828	0.9015	0.4120	0.066*	
H3B	0.6190	1.0005	0.3264	0.066*	
H3C	0.7240	1.0195	0.4606	0.066*	
C4	1.01280 (19)	0.88981 (11)	0.34586 (12)	0.0316 (3)	
N1	0.89312 (16)	0.97171 (9)	0.32919 (11)	0.0323 (3)	
N2	1.1567 (2)	0.87994 (13)	0.27569 (14)	0.0482 (4)	
N3	0.9923 (2)	0.81801 (10)	0.43697 (13)	0.0403 (3)	
O1	0.85571 (15)	1.09547 (8)	0.01761 (9)	0.0385 (3)	
O2	0.74475 (17)	0.94241 (9)	0.09369 (11)	0.0460 (3)	
H1N	1.163 (4)	0.912 (2)	0.208 (3)	0.083 (8)*	
H2N	1.238 (3)	0.8321 (15)	0.2955 (19)	0.051 (5)*	
H3N	1.061 (3)	0.7617 (18)	0.4380 (19)	0.053 (5)*	
H4N	0.914 (2)	0.8210 (14)	0.495 (2)	0.056 (6)*	
H2	0.705 (4)	0.9278 (19)	0.008 (3)	0.084 (7)*	
C11	0.56186 (18)	0.81947 (11)	0.82074 (12)	0.0286 (3)	
C12	0.58163 (18)	0.79173 (11)	0.67945 (12)	0.0284 (3)	
O11	0.45079 (14)	0.76467 (9)	0.87863 (8)	0.0384 (3)	
O12	0.66030 (19)	0.89487 (11)	0.86180 (11)	0.0566 (4)	
O13	0.43397 (14)	0.74502 (9)	0.62504 (9)	0.0385 (3)	
O14	0.72071 (16)	0.81332 (10)	0.62893 (10)	0.0490 (4)	
H13O	0.449 (3)	0.7329 (19)	0.542 (2)	0.071 (6)*	

### Atomic displacement parameters (Å^2^)

**Table:**

	*U*^11^	*U*^22^	*U*^33^	*U*^12^	*U*^13^	*U*^23^
C1	0.0297 (7)	0.0292 (7)	0.0313 (7)	0.0010 (5)	0.0054 (5)	0.0002 (5)
C2	0.0375 (7)	0.0294 (7)	0.0306 (7)	−0.0027 (5)	0.0004 (5)	0.0042 (5)
C3	0.0418 (8)	0.0448 (9)	0.0472 (9)	0.0102 (7)	0.0160 (7)	0.0084 (7)
C4	0.0357 (7)	0.0355 (7)	0.0236 (6)	0.0037 (6)	0.0024 (5)	−0.0009 (5)
N1	0.0341 (6)	0.0333 (6)	0.0303 (6)	0.0037 (5)	0.0064 (5)	0.0058 (4)
N2	0.0442 (8)	0.0637 (9)	0.0384 (8)	0.0205 (7)	0.0147 (6)	0.0151 (7)
N3	0.0507 (8)	0.0353 (7)	0.0364 (7)	0.0121 (6)	0.0128 (6)	0.0077 (5)
O1	0.0498 (6)	0.0348 (5)	0.0308 (5)	−0.0016 (4)	0.0019 (4)	0.0058 (4)
O2	0.0615 (7)	0.0432 (6)	0.0335 (6)	−0.0218 (5)	0.0044 (5)	−0.0033 (5)
C11	0.0283 (6)	0.0346 (7)	0.0230 (6)	0.0007 (5)	0.0013 (5)	−0.0007 (5)
C12	0.0317 (7)	0.0304 (7)	0.0237 (6)	0.0000 (5)	0.0045 (5)	0.0012 (5)
O11	0.0429 (6)	0.0515 (6)	0.0215 (5)	−0.0093 (5)	0.0066 (4)	−0.0017 (4)
O12	0.0687 (8)	0.0683 (8)	0.0339 (6)	−0.0311 (7)	0.0101 (5)	−0.0161 (5)
O13	0.0363 (6)	0.0575 (7)	0.0220 (5)	−0.0070 (5)	0.0042 (4)	−0.0083 (4)
O14	0.0447 (6)	0.0712 (8)	0.0330 (6)	−0.0182 (5)	0.0148 (5)	−0.0060 (5)

### Geometric parameters (Å, °)

**Table:**

C1—O1	1.2060 (17)	C4—N1	1.3296 (18)
C1—O2	1.2943 (17)	N2—H1N	0.82 (3)
C1—C2	1.5090 (19)	N2—H2N	0.85 (2)
C2—N1	1.4493 (17)	N3—H3N	0.85 (2)
C2—H2A	0.9700	N3—H4N	0.86 (2)
C2—H2B	0.9700	O2—H2	0.94 (3)
C3—N1	1.4540 (18)	C11—O12	1.2279 (17)
C3—H3A	0.9600	C11—O11	1.2377 (17)
C3—H3B	0.9600	C11—C12	1.5413 (18)
C3—H3C	0.9600	C12—O14	1.1920 (17)
C4—N2	1.3152 (19)	C12—O13	1.2996 (17)
C4—N3	1.3223 (19)	O13—H13O	0.90 (3)
			
O1—C1—O2	125.59 (13)	C4—N1—C2	121.16 (12)
O1—C1—C2	120.72 (12)	C4—N1—C3	122.23 (12)
O2—C1—C2	113.69 (11)	C2—N1—C3	115.92 (11)
N1—C2—C1	115.09 (11)	C4—N2—H1N	122.7 (19)
N1—C2—H2A	108.5	C4—N2—H2N	118.7 (14)
C1—C2—H2A	108.5	H1N—N2—H2N	118 (2)
N1—C2—H2B	108.5	C4—N3—H3N	117.5 (14)
C1—C2—H2B	108.5	C4—N3—H4N	126.7 (10)
H2A—C2—H2B	107.5	H3N—N3—H4N	115.7 (17)
N1—C3—H3A	109.5	C1—O2—H2	110.9 (15)
N1—C3—H3B	109.5	O12—C11—O11	127.98 (12)
H3A—C3—H3B	109.5	O12—C11—C12	114.67 (12)
N1—C3—H3C	109.5	O11—C11—C12	117.35 (11)
H3A—C3—H3C	109.5	O14—C12—O13	125.56 (12)
H3B—C3—H3C	109.5	O14—C12—C11	121.20 (12)
N2—C4—N3	118.47 (14)	O13—C12—C11	113.24 (11)
N2—C4—N1	121.30 (13)	C12—O13—H13O	110.6 (15)
N3—C4—N1	120.19 (13)		

### Hydrogen-bond geometry (Å, °)

**Table:**

*D*—H···*A*	*D*—H	H···*A*	*D*···*A*	*D*—H···*A*
N2—H1N···O1^i^	0.82 (3)	2.37 (3)	3.094 (2)	148 (2)
N2—H2N···O11^ii^	0.85 (2)	2.08 (2)	2.910 (2)	168 (2)
N3—H3N···O1^iii^	0.85 (2)	2.18 (2)	2.985 (2)	157 (2)
N3—H4N···O14	0.86 (2)	2.04 (2)	2.903 (2)	174 (2)
O2—H2···O12^iv^	0.94 (3)	1.60 (3)	2.538 (2)	173 (2)
O13—H13O···O11^v^	0.90 (3)	1.72 (3)	2.605 (1)	168 (2)

Symmetry codes: (i) −*x*+2, −*y*+2, −*z*; (ii) *x*+1, −*y*+3/2, *z*−1/2; (iii) −*x*+2, *y*−1/2, −*z*+1/2; (iv) *x*, *y*, *z*−1; (v) *x*, −*y*+3/2, *z*−1/2.
